# Effects of Geographical Origin and Tree Age on the Stable Isotopes and Multi-Elements of Pu-erh Tea

**DOI:** 10.3390/foods13030473

**Published:** 2024-02-02

**Authors:** Ming-Ming Chen, Qiu-Hong Liao, Li-Li Qian, Hai-Dan Zou, Yan-Long Li, Yan Song, Yu Xia, Yi Liu, Hong-Yan Liu, Ze-Long Liu

**Affiliations:** 1 Institute of Urban Agriculture, Chinese Academy of Agricultural Sciences, Chengdu National Agricultural Science & Technology Center, Chengdu 610213, China; chenmingming515@163.com (M.-M.C.); liaoqiuhong@caas.cn (Q.-H.L.); zellazou@foxmail.com (H.-D.Z.); a99008191@163.com (Y.-L.L.); seany_lib@163.com (Y.S.); xiayu03@caas.cn (Y.X.); liuyi03@caas.cn (Y.L.); 2 College of Food Science, Heilongjiang Bayi Agricultural University, Daqing 163319, China; qianlili286@163.com; 3 China Food Flavor and Nutrition Health Innovation Center, Beijing Technology and Business University, Beijing 102488, China

**Keywords:** geographical origin, pu-erh tea, tree ages, mineral elements, stable isotope

## Abstract

Pu-erh tea is a famous tea worldwide, and identification of the geographical origin of Pu-erh tea can not only protect manufacture’s interests, but also boost consumers’ confidence. However, tree age may also influence the fingerprints of Pu-erh tea. In order to study the effects of the geographical origin and tree age on the interactions of stable isotopes and multi-elements of Pu-erh tea, 53 Pu-erh tea leaves with three different age stages from three different areas in Yunnan were collected in 2023. The δ^13^C, δ^15^N values and 25 elements were determined and analyzed. The results showed that δ^13^C, δ^15^N, Mg, Mn, Fe, Cu, Zn, Rb, Sr, Y, La, Pr, Nd, Sm, Eu, Gd, Tb, Dy, Ho, Er, Tm, Yb, and Lu had significant differences among different geographical origins (*p* < 0.05). Mn content was significantly influenced by region and tree age interaction. Based on multi-way analysis of variance, principal component analysis and step-wised discriminant analysis, 24 parameters were found to be closely related to the geographical origin rather than tree age, and the geographical origin of Pu-erh tea can be 100.0% discriminated in cross-validation with six parameters (δ^13^C, δ^15^N, Mn, Mg, La, and Tb). The study could provide references for the establishment of a database for the traceability of Pu-erh tea, and even the identification of tea sample regions with different tree ages.

## 1. Introduction

Pu-erh tea, made by large-leaf tea species (*Camellia sinensis var.assamica*), is one of the top ten famous teas in China and is a geographical landmark product of Yunnan [1]. Pu-erh tea is made from Yunnan large-leaf sun-blue maocha. Under the protection of geographical indications, the tea is made by special processing technology, including primary processing (picking, fixing, rolling, and sun-drying) and the blending and pressing of Pu-erh raw tea, the mentation and finishing of Pu-erh ripe tea, as well as the post processing [2]. Exceptionally, plenty of nutrients such as protein, amino acids, carbohydrates, tea polyphenols, and tea pigments have been reported in Pu-erh tea [3]. Pu-erh has multiple health benefits such as anti-cancer [4], antioxidant [5], anti-hypertensive [6], and hypolipidemic properties [7,8,9]. In recent years, the demand for Pu-erh tea has increased rapidly, and consumers have increasingly higher requirements for the quality of Pu-erh tea. The government and enterprises are committed to promoting the development of Pu-erh tea production in the direction of intensification, continuity, technology and digitization.

At present, Pu-erh tea has been listed as a geographical indication product. However, due to its high quality and price, especially raw Pu-erh tea, there are frequently phenomena of substandard quality and fake origin in the market. As a result, there is an urgent need to establish a stable and reliable traceability technology of raw Pu-erh tea’s origin, and to obtain a fresh and authentic identification model by sampling from the original place of origin. Currently, the common traceability methods applied to tea, herbs and spices include chromatographic, spectroscopic and electrochemical methods such as high-performance liquid chromatography [10], gas chromatography mass spectrometry [11], near-infrared spectroscopy [12,13], electronic nose and electronic tongue [14]. In addition, mineral elements [15,16,17] and stable isotopic ratios [18] were also effective fingerprints for geographical traceability. Liu et al. detected four stable isotopes (C, N, H, and O) and 20 elements in green tea samples from different provinces of China, and the correct discrimination rate of green tea samples from different origins was 92.30% [19]. Ni et al. examined the ability to discriminate the geographic origin of green tea by determining the multi-element content and stable isotope signature of flat green tea samples from different origins, combined with the decision tree (DT) method. Under the validation of cross-validation and the “blind” dataset, the prediction accuracy was more than 70.00%, and the discrimination accuracy of green tea from different origins was 90.00% [20].

Among them, the multi-element and stable isotopic ratios were closely related with local geological background (soil [21], water [22], etc.) and environment (temperature [23], precipitation [24], etc.), which proved to be effective tools for geographical traceability. As for the geographical traceability of Pu-erh tea, Zhang et al. analyzed 41 elements in 98 Pu-erh tea samples in Yunnan Province. The results showed that the average concentrations of Fe and Pb in tea samples from Pu-erh were significantly higher than those in other production areas, Mn and Cr were generally higher in Xishuangbanna production area, while Ba and rare earth elements showed higher concentrations in Lancang Pu-erh tea samples. As a result, the geographical origins of Pu-erh tea could be distinguished based on the concentrations of 12 elements combined with chemometric analysis [25]. Li et al. conducted an exploration of the content of eight microelements in raw Pu-erh tea to assess the safety risk related to the storage year [26]. The above literature indicates that researchers mostly focus on mineral element and isotope analysis of tea leaves from different regions, and it is feasible to identify the origin of Pu-erh tea by mass-spectrometric techniques. However, the mineral element and isotopic ratios of tea leaves are not only related to the origin (soil, water, climate) [27,28], but the above parameters may also be influenced by factors such as variety and age of the tree [29,30]. However, there are few reports on the influence of tree age on the fingerprint analysis of fresh Pu ‘er leaves from different geographical origin. Therefore, we will focus on the of different origin, tree age and other factors as a new research idea in this paper.

In this study, the stable isotopic ratios and mineral elemental contents in fresh leaves of Pu-erh tea from different regions and tree ages were collected. The C and N stable isotopes and the elemental contents in tea leaves were analyzed. The indicators mainly influenced by the region and less affected by tree age were screened to establish the robust discriminative model. Based on the above results, the study could lay the theoretical research foundation for the systematic research and database construction work on the origin traceability technology of Pu-erh tea.

## 2. Materials and Methods

### 2.1. Sample Cultivation and Collection

The main production areas (Jinggu County and Ning’er County in Yunnan Pu’er tea City, and Bangdong Township in Lincang City) were selected within the limited area of Geographical Indication Product Pu-erh tea [31]. Two tea gardens were selected in each county. About 200 g of young ‘bud’ leaves (one bud and two leaf) from large-leaf tea species was sampled from tea trees at each sampling site from 26 to 29 March in 2023. Furthermore, specific information including the geographical location information (longitude, latitude, and altitude information) and tree age of Pu-erh tea trees was also recorded. The detailed information is shown in Table 1.

### 2.2. Sample Pretreatment

The leaves were cleaned before drying so as to remove dust and dirt. All tea samples were put into a dryer at 40 °C to obtain a constant weight. Subsequently, the dried samples were finely ground into a uniform powder using a plant crusher. The resulting powder was then passed through a 100-mesh sieve to achieve a homogeneous particle size. All samples were uniformly stored at 4 °C for further analysis.

### 2.3. Multi-Element Analysis

The digestion process for each sample closely followed the methodology outlined in our prior research [1]. Approximately 0.25 g of the homogenized sample was subjected to a 2-h treatment with 6 mL of concentrated HNO_3_ in Teflon digestion vessels. Subsequently, 2 mL of BV-III grade H_2_O_2_ was added to each vessel and allowed to react for 30 min. After the release of nitrogen oxides, the digestion vessels were introduced into a microwave digestion instrument (CEM MARS Xpress, Charlotte, NC, USA) and heated gradually to 180 °C for 40 min.

The ICP-MS operational parameters were as follows: radio frequency power at 1280 W, atomizing chamber temperature at 2 °C. The cooling gas, carrier gas, and auxiliary gas were set at flow rates of 1.47 L min^−1^, 1 L min^−1^, and 1 L min^−1^, respectively. To ensure accuracy, the CRM of tea flour (GBW10016) underwent digestion and determination using the same procedure. All sample determinations were performed in triplicate, with a re-measurement undertaken if the relative standard deviation of internal standard concentration exceeded 5%. Element concentration data were corrected based on dry matter after being adjusted for water content measured before digestion. The quality control (LOD, LOQ, recovery, etc.) of the instrument for the mineral element determination is shown in Table S1.

### 2.4. Stable Carbon and Nitrogen Isotope Analysis

Dry tea samples (0.5–0.6 mg) were carefully weighed into 6.0 mm × 4.0 mm tin capsules and introduced into an elemental analyzer (vario PYRO cube, Elementar Company, Langenselbold, Germany) equipped with an autosampler. Carbon and nitrogen elements within the samples were combusted at 1020 °C, converting them into CO_2_ and NOx gases. Subsequently, the NOx was reduced to N_2_ through a copper wire at 600 °C before entering an isotope ratio mass spectrometer (IsoPrime100, Isoprime Company, Stockport, UK) via a Conflo III dilutor.

The final stable isotope ratios are expressed as δ notation relative to international standard (Vienna Pee Dee Belimnite (VPDB) for carbon, atmospheric nitrogen (AIR) for nitrogen), according to the following equation:

δ (‰) = (R_sample_/R_standard_ − 1) × 1000,

where δ (‰) represents the δ^13^C and δ^15^N values, and R is the ratio of ^13^C/^12^C or ^15^N/^14^N.

For scale normalization and quality assurance, tea samples were analyzed together with reference materials including USGS40 (L-glutamic acid; δ^13^C_VPDB_ = −26.389‰, δ^15^N_air_ = −4.5‰) and urea (δ^13^C_VPDB_ = −43.26‰, δ^15^N_air_ = −0.56‰) for δ^13^C and δ^15^N values. Each sample was analyzed three times. The instrumental precision for stable isotope ratio measurements based on the reference materials was ≤0.2‰ for δ^13^C values, ≤0.2‰ for δ^15^N values, ≤3‰ values, respectively.

### 2.5. Statistical Analysis

The statistical analyses of the data, including one-way analysis of variance (one-way ANOVA), multiway analysis of variance (multiway ANOVA), principal component analysis (PCA) and linear discriminant analysis (LDA), were carried out with SPSS for Windows version 22.0 (SPSS Inc., Chicago, IL, USA).

One-way ANOVA was applied to elements to test whether the differences in average elemental values are related to considered geographical origins. With post-hoc analysis conducted using either Dunnett’s or Tukey’s test for multiple comparisons according to the result of Bartlett’s test for equal variances. Multiway ANOVA was applied was to quantify the contributions of geographical origin, tree age and their interactions (three factors) to the total variance in element levels. A factor with a larger ratio of relative variance indicates the greater influence relative to the other factors.

Principal component analysis (PCA) is used to transform a set of correlated variables into a set of uncorrelated principal components (PCs) that explain the greatest possible amount of variation in the data, and to provide a comprehensive data visualization [32]. Upon applying PCA to the analytical data for three geographical origins, tea samples could be preliminarily clustered (the first four PCs). In addition, we used Fisher’s linear discrimination analysis (LDA) to assess the effectiveness of the elements for the identification of tea origin traceability. Linear discriminant analysis (LDA) is a supervised procedure that maximizes the variances between categories and minimizes the variances within categories by creating new variables (discriminant functions). The reliability of the discriminant model was also verified by the cross-validation method (leave-one-out method).

## 3. Results

### 3.1. Comparison of Isotopic Ratios and Mineral Contents from Different Regions

**Figure 1:**
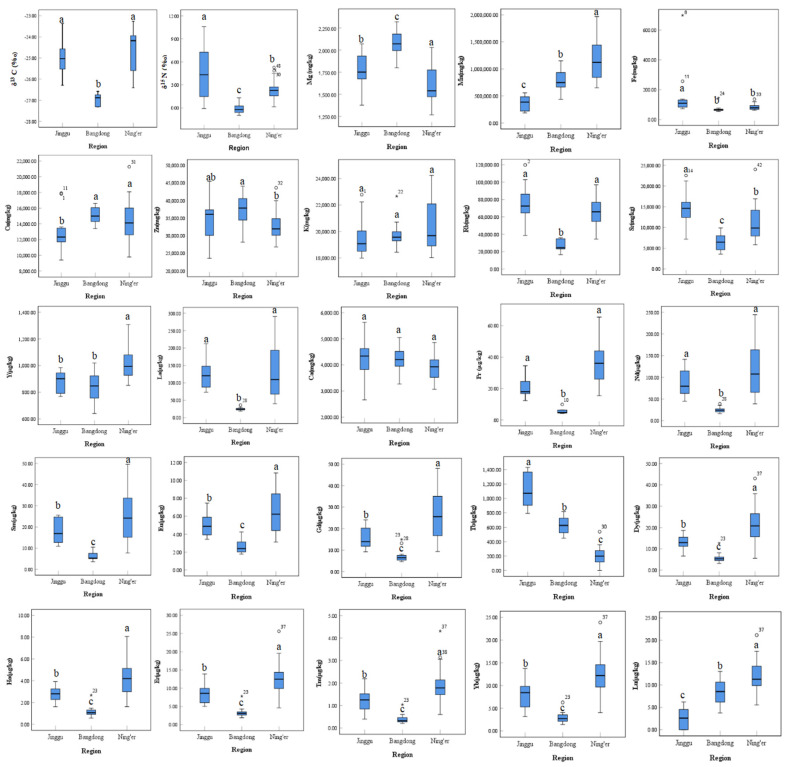
Box plots of stable isotope ratio and mineral content in Pu-erh tea in different regions. ^a–c^ in the same row indicated that there are significant differences among regions at *p* < 0.05 level. Note: ◦ indicates outliers, and the number in the upper right corner represents the number of in-dividuals that are outliers; * denotes outliers, the number in the upper right corner represents the number of individuals that are outliers.

The mean values and standard deviations of mineral element contents in Pu-erh tea samples from different regions are shown in Table 2. The mineral elements (Mg, Mn, Fe, Cu, Zn, Rb, Sr, Y, La, Pr, Nd, Sm, Eu, Gd, Tb, Dy, Ho, Er, Tm, Yb and Lu) and isotopes (δ^15^N and δ^13^C) had significant differences among different geographical origins (*p* < 0.05). The mineral elements (Mg, Mn, Fe, Cu, Rb, Sr, La, Pr, Nd, Sm, Eu, Gd, Tb, Dy, Ho, Er, Tm, Yb and Lu) and isotopes (δ^15^N and δ^13^C) had significant differences between the two geographical origins of Jinggu and Bangdong (*p* < 0.01). The mineral elements (Mn, Y, Gd, Tb, Dy, Ho, Er, Tm, Yb and Lu) had significant differences between the two geographical origins of Jinggu and Ning’er (*p* < 0.01). The mineral elements (Mg, Mn, Rb, Sr, Y, La, Pr, Nd, Sm, Eu, Gd, Tb, Dy, Ho, Er, Tm, Yb and Lu) and isotopes (δ^15^N and δ^13^C) had significant differences between the two geographical origins of Bangdong and Ning’er (*p* < 0.01). Specifically, the highest elemental content of K was found in Ning’er tea samples, and the Fe, Sr, Tb contents and δ^15^N were significantly higher in Pu-erh tea samples from Jinggu than in other regions, the elemental contents of Mg, K and Ca were higher in Pu-erh tea samples from Bangdong, while the δ^13^C value and the elemental contents of Mg, Mn, Y, Sm, Eu, Gd, Dy, Ho, Er, Tm, Yb, and Lu were significantly higher in Pu-erh tea samples from Ning’er than in other regions. Box plots of stable isotope ratios and mineral contents in Pu-erh tea in different regions are shown in Figure 1.

The mean values and standard deviations of mineral element contents in deep soil (30–60 cm) from different regions are shown in Table 3. The mineral elements (Mg, K, Ca, Mn, Fe, Cu, Zn, Rb, Sr, Y, La, Pr, Nd, Sm, Eu, Gd, Tb, Dy, Ho, Er, Tm, Yb and Lu) had significant differences among different geographical origins (*p* < 0.05). The mineral elements (Mg, K, Mn, Zn, Rb, Y, Pr, Nd, Sm, Eu, Gd, Tb, Dy, Ho and Er) had significant differences between Jinggu and Bangdong (*p* < 0.01). The mineral elements (Mg, K, Mn, Fe, Cu, Zn, Rb, Sr, Y, Eu, Tb, Dy, Ho, Er, Tm, Yb and Lu) had significant differences between Jinggu and Ning’er (*p* < 0.01). The mineral elements (Mg, Fe, Cu, Zn, Rb and Sr) had significant differences between Bangdong and Ning’er (*p* < 0.01). In summary, the trends of the elements (Mn, Rb, Tb and Dy) in the soil in the three geographic regions were consistent with those in Pu-erh tea.

**Figure 2:**
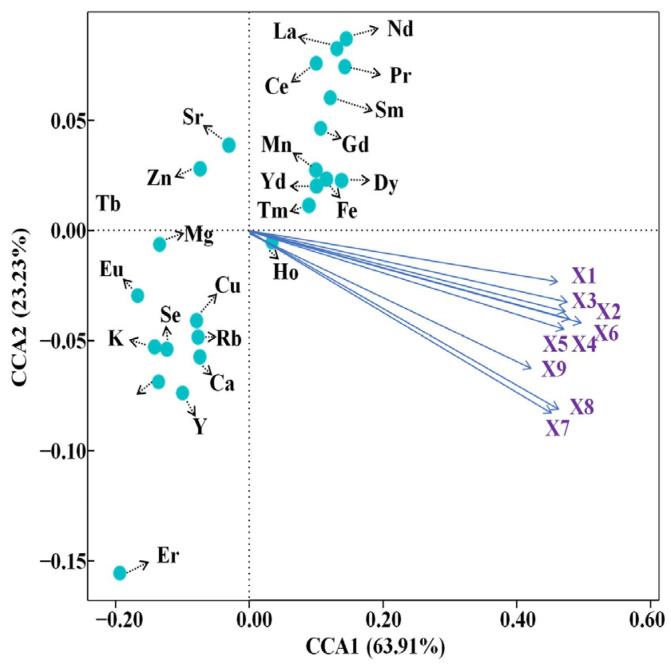
Canonical correlation analysis plot.

As can be seen from Table 4, the canonical correlation analysis (CCA) extracted a total of 9 groups of typical variables, of which 7 groups of typical variables had a correlation coefficient of 0.317, and 8 groups of typical variables had a correlation coefficient of 0.111. As can be seen in Figure 2, when CCA1 was taken as a benchmark, the contents of Nd, La, Pr, Sm, Gd, Mn, Yb, Dy, Fe, Tm, and Ce were positively correlated with soil elemental content, and Eu, Cu, Rb, K, Se, Ca, Tb, and Y were negatively correlated with soil elemental content, of which the content of Ho in tea was weakly correlated with soil elemental content; when CCA2 was taken as a benchmark, the content of Zn, and Sr in tea was positively correlated with soil elemental content, Eu, Cu, Rb, K, Se, Ca, Tb, and Y were positively correlated with soil elemental content, and Eu, Cu, Rb, K, Se, Ca, Tb, and Y were positively correlated with soil elemental content. The contents of Eu, Cu, Rb, K, Se, Ca, Tb, and Y were negatively correlated with the soil element contents, and the content of Mg in tea was weakly negatively correlated with the soil element contents. At the same time, the soil characteristics of the different regions (pH, EC, etc.) are shown in Table 5. Among them, the highest pH value was found in Bangdong, which was significantly different from the other two regions (*p* < 0.01). The lowest EC value was found in Ning’er, which was highly significantly different from the other two regions (*p* < 0.01).

### 3.2. Comparison of Isotopic Ratios and Mineral Contents from Different Tree Ages

The mean values and standard deviations of mineral element contents in Pu-erh tea samples of different ages are shown in Table 6. In addition to Zn, other mineral elements (Mg, K, Ca, Mn, Fe, Cu, Rb, Sr, Y, La, Pr, Nd, Sm, Eu, Gd, Tb, Dy, Ho, Er, Tm, Yb, and Lu) and isotopes (δ^13^C and δ^15^N) did not show any significant differences (*p* > 0.05) among tree ages. Among them, the elemental content of Zn was higher in Pu-erh tea samples aged 100~200 than in other ages. Specifically, the elemental differences among tree ages for each region are shown in Table 7. In the region of Bangdong, significant differences (*p* < 0.05) were found for δ^13^C and the contents of Ca, Zn, Ho, and Yb in tea leaves among different tree ages. However, the contents of Ca, Pr, Nd, Sm, Eu and Gd in tea leaves had significant differences in the region of Ning’er (*p* < 0.05).

### 3.3. Multi-Way Analysis of Variance for Stable Isotopic Ratios and Elements

A combined analysis of variance across three regions and three tree ages was performed using the general linear model (GLM) procedure of SPSS (Table 8). Regions and tree age were considered as fixed factors, and the effects were portioned into different sources, such as region (R), age (A), and region × age (R × A). In total, the stable isotope values (δ^15^N and δ^13^C) and the mineral contents (Mg, Mn, Rb, Sr, Y La, Pr, Nd, Sm, Eu, Gd, Tb, Dy, Ho, Er, Tm, Yb, and Lu) in Pu-erh tea were highly significantly influenced by region (*p* < 0.01), the contents of Fe and Zn in Pu-erh tea were significantly influenced by region (*p* < 0.05), whereas R × A had significant effects on Mn content (*p* < 0.01).

### 3.4. Principal Component Analysis of Isotope Ratios and Mineral Content of Pu-erh Tea from Different Regions

**Figure 3:**
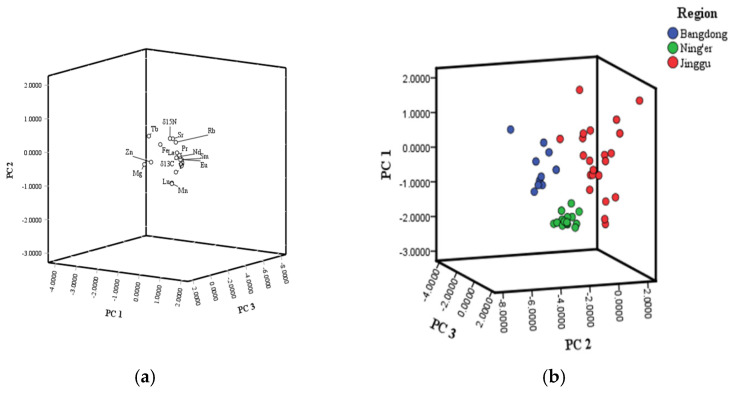
Discriminant function scores of Pu-erh tea of different regions. (**a**) The Pu-erh tea samples from different origin sources were plotted using principal component scores; (**b**) The scores of Pu-erh tea from different origin sources were plotted using the scores of PC1, PC2, and PC3.

Through the above effects of region, tree age and their interaction on the isotope ratios and mineral contents of Pu-erh tea, 22 characteristic mineral elements related to the regions, including Mg, Mn, Fe, Zn, Rb, Sr, Y, La, Pr, Nd, Sm, Eu, Gd, Tb, Dy, Ho, Er, Tm, Yb, Lu, δ^13^C and δ^15^N were screened. The screened 22 characteristic mineral elements were subjected to principal component analysis of different regions, and the results are shown in Table S2. Four principal components were obtained with a cumulative contribution of 82.54%. Principal component 1 mainly contains δ^13^C, δ^15^N, Fe, Cu, Sr, Y, La, Pr, Nd, Sm, Eu, Gd, Tb, Ho, Er, Tm, Yb and Lu, and the contribution rate is 57.76%, principal component 2 mainly contains δ^15^N, Cu, Sr, Y, Pr, Nd, Sm and Dy element information, and the contribution rate is 13.43%, principal component 3 mainly contains the information of δ^15^N and Mg, Cu, Rb, Pr, Nd, Sm, Eu, Tb and Dy elements, and the contribution rate is 6.76%, principal component 4 mainly contains δ^13^C, Fe, Cu, Rb, Sr and La information with a contribution rate of 4.60%. The four most important variables were Nd, Sm, Eu, Gd, Tb, Ho, Er, Tm, Yb and Lu in PC1, and δ^15^N, Sr, Y and Dy in PC2, and Mg in PC3, respectively (Figure 3a). Meanwhile, three origin samples (Ning’er, Jinggu, Bangdong) were correctly distinguished. And they are distributed in different spatial regions. It shows that the selected mineral elements and stable isotope origin traceability fingerprint can be used to distinguish Pu-erh tea from different origins (Figure 3b). The Pu-erh tea samples from three geographical origins were distributed in different spatial distributions. The results indicated that these elements could be used for the identification of the geographical origin of Pu-erh tea.

### 3.5. Discriminant Analysis of Isotope Ratio and Mineral Element Content of Pu-erh Tea from Different Regions

Stepwise discriminant analysis was conducted on Mg, Mn, Fe, Zn, Rb, Sr, Y, La, Pr, Nd, Sm, Eu, Gd, Tb, Dy, Ho, Er, Tm, Yb, Lu, δ^13^C and δ^15^N, and seven indicators were selected to establish the discriminant model. The cross-validation model was also used to obtain the classification results of Pu-erh tea samples from three regions, as shown in Table S3. Based on the discriminant model using the screened elements, the classification results showed that both original and cross-validation of correct discriminant rates could reach 100%. The result indicated that a discriminative model consisting of these elements was fully capable of correctly discriminating samples.

The discrimination model formula is as follows:

Y_Bangdong_ = −44.943 δ^13^C + 0.081 δ^15^N + 0.42 Mg + 2.863 × 10^−5^ Mn + 8.186 × 10^−6^ Rb + 0.175 La + 0.101 Tb − 675.771

Y_Ning’er_ = −48.217 δ^13^C − 3.003 δ^15^N + 0.054 Mg + 4.555 × 10^−5^ Mn − 3.021 × 10^−4^ Rb + 0.129 La + 0.068 Tb − 739.961

Y_Jinggu_ = −45.218 δ^13^C − 3.404 δ^15^N + 0.031 Mg + 4.504 × 10^−5^ Mn − 1.039 × 10^−4^ Rb + 0.220 La + 0.034 Tb − 621.148

**Figure 4:**
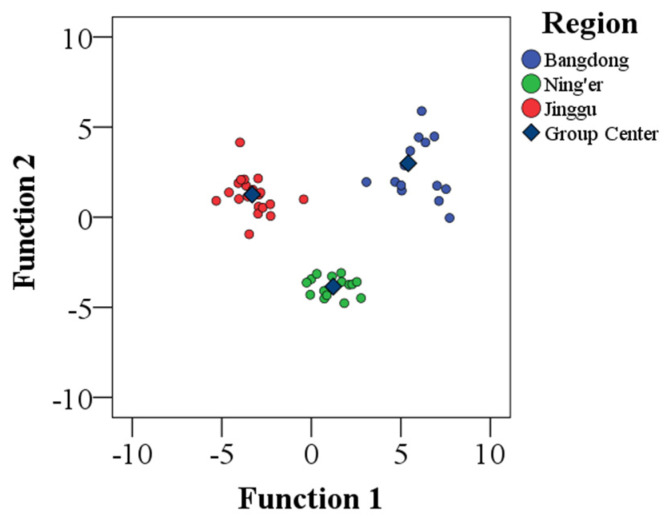
Discriminant function score map of Pu-erh tea from different regions.

As shown in Figure 4, the distribution of Pu-erh tea from different regions was obtained. As was seen from the figure, the Pu-erh tea samples from the three regions were completely distinguished and located in different spaces, and there was a certain spatial range between the regions, indicating that the selected indicators related to the region were accurate and effective.

As can be seen from Table 9, the model was validated by determining the indicators (δ^13^C, δ^15^N, Mg, Mn, Rb, La, and Tb) of nine Pu-erh tea samples from Bangdong. All the samples were correctly discriminated, and a correct discrimination rate of 100.00% was obtained.

## 4. Discussion

In our study, there were significant differences in stable isotopes among the three regions. The lowest δ^13^C value was found in Pu-erh tea leaves from the Bangdong region, while no significant difference was found between the other two regions. The δ^13^C value in tea leaves was shown as follows: Ning’er > Jinggu > Bangdong, while the tendency of latitude for the three regions was the same, which indicated that the δ^13^C values in tea leaves increased with the higher latitude, which is consistent with the previous results [33]. Generally, tea areas at low latitudes have high average annual temperatures and receive more light radiation on the surface of the ground. Pu-erh tea, as a C_3_ plant, utilizes the Calvin photosynthetic pathway for CO_2_ assimilation. The δ^13^C values in plants have been found to correlate with the ratio of intercellular CO_2_ and CO_2_ from the surrounding environment (P_i_/P_a_) [34]. This correlation reflects the variability in CO_2_ sources (both concentrations and δ^13^C values) across different regions. Specifically, higher latitude is often associated with higher atmospheric CO_2_ concentrations, which in turn results in variations in physiological processes and δ^13^C values within Pu-erh tea. In addition, the δ^13^C values of Pu-erh tea samples with different geographical origins ranged from −24.58‰ to −27.15‰, falling in the range of C_3_ plants (−34‰ to −24‰) [35], and the result was agreed with a previous report in which δ^13^C values for Chinese green tea varied between −28.5‰ and −24.5‰ [36]. The observed variation in δ^15^N value was found in Pu-erh tea from the Bangdong region, while there was no significant difference in values. Numerous investigations have showcased the correlation between fertilizer application in agricultural techniques and the δ^15^N levels in plants. Traditionally, synthetic nitrogen-based fertilizers exhibit nitrogen isotope values ranging from −4‰ to 4‰, while organic fertilizers generally manifest higher δ^15^N values and exhibit a considerably broader spectrum (2–30‰) than their synthetic counterparts [37]. It is worth mentioning that distinct synthetic nitrogen fertilizers may also display varying δ^15^N values.

In addition, the contents of mineral elements such as Rb, La, Pr, and Nd were lower in Pu-erh tea leaves from the Bangdong region, while no significant difference was found between the other two regions. There were significant differences in the contents of mineral elements (Fe) in Pu-erh tea leaves in the Jinggu region compared with other regions. This might be because the mineral elements in plants were closely related to the mineral contents in the regional soil [38]. The mineral contents in soils were mainly influenced by conditions such as soil-forming parent material, soil pH, climate, and precipitation, which formed the specific elemental fingerprints among different regions. The characteristics of the “soil-plant” system led to variations in the composition of trace elements in plant tissues. At the same time, compared with the previous mineral elements of Pu-erh tea, especially the Mg values of Pu-erh tea samples with different geographical origins ranged from 1797.00 to 2273.50 mg/kg, agreeing with a previous report in which Mn values for Pu-erh tea varied between 381.65 to 878.15 mg/kg [39]. For Pu-erh tea from Yunnan Province, the total amount of Eu (0.010 ± 0.002 μg/g) was close to a previous report in which the content of Eu was 6.49 ± 2.30 μg/kg, and the Yb content (0.019 ± 0.006 μg/g) was also similar with the previous report, with the Yb content was 12.25 ± 4.66 μg/kg [40]. Furthermore, the mineral element content (e.g., Zn) in Pu-erh tea is also influenced by the age of the tea trees. Differences in Zn content can arise as a result of variations in the growing environment of tea trees of different ages, given that Zn constitutes one of the vital trace elements needed for plant growth and development. However, additional comprehensive investigations are required to establish the pattern of change in the content of other mineral elements across different ages of Pu-erh tea trees.

Although previous studies have obtained good discrimination for the geographical origins of tea by using the stable isotope techniques or mineral element techniques [41,42], the combination of two techniques was used for the first time to effectively identify the origin of fresh Pu-erh tea, obtaining a high overall correct classification rate (100.0%) and cross-validation rate (100.0%).

## 5. Conclusions

In this study, δ^13^C and δ^15^N values and 24 elemental contents of Pu-erh tea samples from different regions and tree ages were comprehensively analyzed. Based on the multi-way analysis of variance, only those significantly influenced by region were screened to better distinguish between samples of different geographical origins. Using the screened elements, step-wised discriminant analysis with 100.0% correct discrimination and 100.0% cross-validation was obtained, and a discriminant model was established with only six parameters (δ^13^C, δ^15^N, Mn, Mg, La, and Tb), indicating that the elements screened in relation to origin were accurate and effective. Therefore, it is technically feasible to screen the effective stable isotope and mineral elements to discriminate the origin of Pu-erh tea. In addition, the study could also provide a reference for the establishment of a database for the traceability of Pu-erh tea’s geographical origin. Because the tree age of 100 years is a variable, the data should be available for identification in the second year. However, it requires resampling for validation. Two tea plantations were chosen from each region, which were representative to a certain extent but still this is not many. Therefore, further expansion of the production areas under study is needed.

## Figures and Tables

**Table 1:** The sample numbers, location, and tree age of tea geographical origins.

Region	Number of Samples	N Latitude (deg)	E Longitude (deg)	Altitude (m)	Tree Age (Year)
Jinggu	24	23.7227	100.6877	1842–1901	20~100 (12), 100~200 (6), >200 (6)
Bangdong	14	23.9374–23.9397	100.3532–100.3562	1633–1739	20~100 (8), 100~200 (3), >200 (3)
Ning’er	15	23.2548	101.0822	1614	20~100 (5), 100~200 (5), >200 (5)

**Table 2:** Stable isotope ratios and mineral contents in Pu-erh tea from different regions.

Element	Jinggu	Bangdong	Ning’er
δ^13^C (‰) *	−25.16 ± 0.83 ^a^	−26.77 ± 0.61 ^b^	−24.82 ± 1.16 ^a^
δ^15^N (‰) **	4.06 ± 3.35 ^a^	−0.11 ± 0.66 ^c^	2.39 ± 1.30 ^b^
Mg (mg/kg) **	1776.176 ± 184.688 ^b^	2077.253 ± 152.236 ^c^	1601.886 ± 201.680 ^a^
K (mg/kg)	19,617.81 ± 1591.58 ^a^	19,701.25 ± 1035.70 ^a^	20,368.84 ± 1833.42 ^a^
Ca (mg/kg)	4193.70 ± 820.56 ^a^	4184.00 ± 486.66 ^a^	3864.72 ± 469.04 ^a^
Mn (mg/kg) **	376 ± 139 ^c^	790 ± 219 ^b^	1159 ± 359 ^a^
Fe (mg/kg) *	155 ± 164 ^a^	70 ± 22 ^b^	84 ± 20 ^b^
Cu (mg/kg) *	12.7 ± 2.5 ^b^	15.2 ± 1.1 ^a^	14.3 ± 2.5 ^a^
Zn (mg/kg) *	34.7 ± 5.9 ^ab^	37.3 ± 4.8 ^a^	32.9 ± 4.2 ^b^
Rb (mg/kg) *	75 ± 21 ^a^	28 ± 6 ^b^	65 ± 17 ^a^
Sr (mg/kg) **	14 ± 5 ^a^	7 ± 2 ^c^	11 ± 4 ^b^
Y (μg/kg) *	876.96 ± 79.77 ^b^	843.16 ± 120.60 ^b^	1020.33 ± 125.51 ^a^
La (μg/kg) *	119.86 ± 38.80 ^a^	25.08 ± 4.42 ^b^	123.80 ± 68.58 ^a^
Pr (μg/kg) *	24 ± 8 ^a^	6 ± 2 ^b^	29 ± 14 ^a^
Nd (μg/kg) *	88.38 ± 30.35 ^a^	24.53 ± 6.16 ^b^	115.51 ± 54.43 ^a^
Sm (μg/kg) **	18 ± 6 ^b^	6 ± 2 ^c^	25 ± 12 ^a^
Eu (μg/kg) **	5.0 ± 1.2 ^b^	2.7 ± 0.8 ^c^	6.5 ± 2.3 ^a^
Gd (μg/kg) **	16 ± 5 ^b^	7 ± 3 ^c^	26 ± 11 ^a^
Tb (μg/kg) **	1122.8 ± 238.5 ^a^	630.6 ± 124.7 ^b^	219.6 ± 110.1 ^c^
Dy (μg/kg) **	13 ± 4 ^b^	6 ± 2 ^c^	21 ± 9 ^a^
Ho (μg/kg) **	2.7 ± 0.7 ^b^	1.2 ± 0.5 ^c^	4.3 ± 1.6 ^a^
Er (μg/kg) **	9 ± 3 ^b^	3 ± 1 ^c^	13 ± 5 ^a^
Tm (μg/kg) **	1.2 ± 0.5 ^b^	0.4 ± 0.2 ^c^	1.9 ± 0.8 ^a^
Yb (μg/kg) **	8 ± 3 ^b^	3 ± 1 ^c^	13 ± 5 ^a^
Lu (μg/kg) **	3.6 ± 1.8 ^c^	8.5 ± 2.9 ^b^	11.8 ± 3.9 ^a^

Data are shown as the mean ± standard deviation. ^a–c^ in the same row indicated that there are significant differences among regions at *p* < 0.05 level. * means significant difference (*p* < 0.05), ** means highly significant difference (*p* < 0.01).

**Table 3:** Mineral contents in deep soil (30–60 cm) from different regions.

Element	Jinggu	Bangdong	Ning’er
Mg (μg/kg) *	4973.044 ± 1006.566 ^a^	2381.777 ± 168.522 ^b^	1535.505 ± 76.886 ^b^
K (mg/kg) *	27.28 ± 2.41 ^a^	10.85 ± 0.08	11.55 ± 0.27 ^b^
Ca (μg/kg) *	66.26 ± 22.42 ^a^	51.38 ± 5.59 ^ab^	37.26 ± 1.24 ^b^
Mn (μg/kg) **	1264 ± 46 ^b^	1470 ± 40 ^a^	341 ± 26 ^c^
Fe (mg/kg) *	38 ± 5 ^a^	32 ± 1 ^a^	17 ± 1 ^b^
Cu (μg/kg) *	57.2 ± 7.8 ^a^	49.3 ± 1.6 ^a^	18.4 ± 0.3 ^b^
Zn (μg/kg) **	156.1 ± 14.7 ^a^	96.9 ± 3.1 ^b^	42.9 ± 2.1 ^c^
Rb (μg/kg) **	134 ± 13 ^a^	49 ± 4 ^c^	82.36 ± 2.04 ^b^
Sr (μg/kg) **	28 ± 4 ^b^	36 ± 0 ^a^	20 ± 1 ^c^
Y (μg/kg) *	18.18 ± 1.35 ^a^	11.66 ± 0.85 ^b^	9.68 ± 1.80 ^b^
La (μg/kg) *	40.03 ± 2.05 ^a^	27.05 ± 1.64 ^b^	31.82 ± 7.64 ^ab^
Pr (μg/kg) *	9 ± 0 ^a^	5 ± 0 ^b^	6 ± 2 ^b^
Nd (μg/kg) *	34.81 ± 1.35 ^a^	18.09 ± 1.27 ^b^	20.85 ± 7.20 ^b^
Sm (μg/kg) *	7 ± 0 ^a^	3 ± 0 ^b^	4 ± 2 ^b^
Eu (μg/kg) **	0.8 ± 0.1 ^a^	0.5 ± 0.0 ^b^	0.3 ± 0.0 ^c^
Gd (μg/kg) *	6 ± 0 ^a^	2 ± 0 ^b^	3 ± 1 ^b^
Tb (μg/kg) *	0.7 ± 0.1 ^a^	0.3 ± 0.0 ^b^	0.4 ± 0.1 ^b^
Dy (μg/kg) *	4 ± 0 ^a^	2 ± 0 ^b^	2 ± 1 ^b^
Ho (μg/kg) *	0.7 ± 0.1 ^a^	0.4 ± 0.0 ^b^	0.3 ± 0.1 ^b^
Er (μg/kg) *	2 ± 0 ^a^	1 ± 0 ^b^	1 ± 0 ^b^
Tm (μg/kg) **	0.2 ± 0.0 ^a^	0.2 ± 0.0 ^b^	0.1 ± 0.0 ^c^
Yb (μg/kg) **	2 ± 0 ^a^	1 ± 0 ^b^	1 ± 0 ^c^
Lu (μg/kg) **	0.2 ± 0.0 ^a^	0.1 ± 0.0 ^b^	0.1 ± 0.0 ^c^

Data are shown as the mean ± standard deviation. ^a–c^ in the same row indicated that there are significant differences among regions at *p* < 0.05 level. * means significant difference (*p* < 0.05), ** means highly significant difference (*p* < 0.01).

**Table 4:** The canonical correlation coefficients.

Canonical Variable	Correlation Coefficient
1	−0.077
2	−0.150
3	−0.069
4	−0.255
5	−0.246
6	−0.215
7	0.317
8	0.111
9	0.371

**Table 5:** The soil characteristics of the different regions (pH, EC, etc.).

Region	Bangdong	Jinggu	Ning’er
pH	5.48 ± 0.15 ^a^	5.07 ± 0.17 ^b^	5.03 ± 0.06 ^b^
EC (μs/cm)	46.98 ± 0.93 ^a^	34.99 ± 5.97 ^b^	24.21 ± 5.64 ^c^

^a–c^ in the same row indicated that there are significant differences among regions at *p* < 0.05 level.

**Table 6:** Stable isotopes and mineral contents in Pu-erh tea from different tree ages.

Element	20~100	100~200	>200
δ^13^C (‰)	−25.19 ± 1.27 ^a^	−25.56 ± 1.07 ^a^	−25.86 ± 1.37 ^a^
δ^15^N (‰)	1.82 ± 2.30 ^a^	2.43 ± 2.93 ^a^	2.36 ± 2.43 ^a^
Mg (mg/kg)	1758.798 ± 257.603 ^a^	1799.159 ± 335.310 ^a^	1808.026 ± 233.497 ^a^
K (mg/kg)	20,348.89 ± 1777.85 ^a^	19,778.20 ± 1233.41 ^a^	19,528.80 ± 1504.73 ^a^
Ca (mg/kg)	4109.37 ± 567.86 ^a^	3826.25 ± 757.32 ^a^	4137.37 ± 431.45 ^a^
Mn (μg/kg)	821 ± 481 ^a^	889 ± 363 ^a^	855 ± 400 ^a^
Fe (mg/kg)	1171 ± 124 ^a^	86 ± 50 ^a^	80 ± 20 ^a^
Cu (mg/kg)	13.9 ± 2.5 ^a^	15.2 ± 2.5 ^a^	13.4 ± 1.7 ^a^
Zn (mg/kg) *	34.4 ± 4.7 ^ab^	37.1 ± 5.9 ^a^	32.4 ± 4.0 ^b^
Rb (mg/kg)	62 ± 25 ^a^	51 ± 23 ^a^	55 ± 25 ^a^
Sr (mg/kg)	10 ± 4 ^a^	112 ± 6 ^a^	12 ± 5 ^a^
Y (μg/kg)	935.15 ± 137.55 ^a^	942.68 ± 126.28 ^a^	916.89 ± 159.38 ^a^
La (μg/kg)	109.72 ± 68.93 ^a^	68.80 ± 46.51 ^a^	94.24 ± 74.79 ^a^
Pr (μg/kg)	25 ± 16 ^a^	16 ± 9 ^a^	20 ± 14 ^a^
Nd (μg/kg)	95.70 ± 61.48 ^a^	61.80 ± 35.75 ^a^	79.98 ± 55.41 ^a^
Sm (μg/kg)	21 ± 13 ^a^	13 ± 7 ^a^	17 ± 11 ^a^
Eu (μg/kg)	5.4 ± 2.6 ^a^	4.3 ± 1.8 ^a^	5.1 ± 2.3 ^a^
Gd (μg/kg)	20 ± 13 ^a^	14 ± 7 ^a^	18 ± 11 ^a^
Tb (μg/kg)	638.3 ± 478.3 ^a^	520.7 ± 306.6 ^a^	560.2 ± 351.2 ^a^
Dy (μg/kg)	16 ± 10 ^a^	12 ± 6 ^a^	146 ± 9 ^a^
Ho (μg/kg)	3.3 ± 2.0 ^a^	2.5 ± 1.2 ^a^	3.0 ± 1.8 ^a^
Er (μg/kg)	10 ± 6 ^a^	8 ± 4 ^a^	9 ± 5 ^a^
Tm (μg/kg)	1.4 ± 1.0 ^a^	1.1 ± 0.7 ^a^	1.3 ± 0.9 ^a^
Yb (μg/kg)	9 ± 6 ^a^	7 ± 4 ^a^	9 ± 5 ^a^
Lu (μg/kg)	9.9 ± 5.4 ^a^	7.9 ± 3.3 ^b^	9.2 ± 4.0 ^c^

Data are shown as the mean ± standard deviation. ^a–c^ in the same row indicated that there are significant differences among tree ages at *p* < 0.05 level. * means significant difference (*p* < 0.05).

**Table 7:** Stable isotopes and mineral content of Pu-erh tea of different tree ages in each origin.

Element	Jinggu			Bangdong			Ning’er		
	20~100	100~200	>200	20~100	100~200	>200	20~100	100~200	>200
δ^13^C (‰) *	−24.99 ± 0.90 ^a^	−25.46 ± 0.33 ^a^	−25.33 ± 1.11 ^a^	−26.97 ± 0.34 ^b^	−26.19 ± 0.53 ^a^	−27.15 ± 0.50 ^b^	−24.58 ± 1.04 ^a^	−25.09 ± 1.43 ^a^	−25.04 ± 1.22 ^a^
δ^15^N (‰)	2.53 ± 3.20 ^a^	6.82 ± 3.29 ^a^	5.37 ± 1.67 ^a^	−0.53 ± 0.50 ^a^	0.21 ± 0.88 ^a^	−0.01 ± 0.38 ^a^	2.33 ± 1.33 ^a^	2.08 ± 0.88 ^a^	2.83 ± 1.65 ^a^
Mg (mg/kg)	1801.02 ± 210.65 ^a^	1731.73 ± 184.49 ^a^	1754.36 ± 161.95 ^a^	2022.54 ± 196.35 ^a^	2146.70 ± 150.99 ^a^	2062.52 ± 98.59 ^a^	1620.76 ± 220.96 ^a^	1543.25 ± 241.60 ^a^	1622.79 ± 125.77 ^a^
K(mg/kg)	19,745.13 ± 1778.10 ^a^	19,765.25 ± 1961.40 ^a^	19,130.87 ± 1048.56 ^a^	19,640.30 ± 794.35 ^a^	20,228.30 ± 1420.24 ^a^	19,235.15 ± 694.88 ^a^	21,046.64 ± 1905.34 ^a^	19,409.59 ± 661.17 ^a^	19,972.49 ± 2154.85 ^a^
Ca (mg/kg) *	4573.75 ± 567.83 ^a^	3250.35 ± 1008.22 ^b^	4123.57 ± 558.13 ^a^	3886.28 ± 424.46 ^b^	4532.52 ± 350.73 ^a^	4133.20 ± 509.79 ^ab^	3892.74 ± 449.67 ^ab^	3525.65 ± 424.20 ^b^	4147.75 ± 388.87 ^a^
Mn (mg/kg)	345 ± 147,577 ^a^	390 ± 162,990 ^a^	443 ± 106,601 ^a^	2 ± 196 ^a^	2 ± 151 ^a^	2 ± 99 ^a^	1207 ± 362 ^a^	1130 ± 299 ^a^	1093 ± 451 ^a^
Fe (mg/kg)	182 ± 210 ^a^	138 ± 103 ^a^	100 ± 32 ^a^	76 ± 39 ^a^	66 ± 7 ^a^	68 ± 4 ^a^	90 ± 25 ^a^	76 ± 10 ^a^	80 ± 15 ^a^
Cu (mg/kg)	12.1 ± 2.6 ^a^	14.5 ± 3.1 ^a^	12.5 ± 0.9 ^a^	15.6 ± 1.1 ^a^	15.6 ± 0.8 ^a^	14.4 ± 0.9 ^a^	14.5 ± 2.3 ^a^	15.1 ± 3.3 ^a^	13.1 ± 2.1 ^a^
Zn (mg/kg) *	35.5 ± 4.8 ^a^	34.9 ± 11.0 ^a^	32.4 ± 3.7 ^a^	37.1 ± 3.6 ^ab^	41.7 ± 2.2 ^a^	33.0 ± 4.1 ^b^	32.6 ± 4.7 ^a^	34.5 ± 2.5 ^a^	31.9 ± 4.6 ^a^
Rb (mg/kg)	77 ± 21 ^a^	62 ± 24 ^a^	82 ± 18 ^a^	26 ± 5 ^a^	27 ± 8 ^a^	32 ± 5 ^a^	67 ± 17 ^a^	65.477 ± 15 ^a^	60 ± 21 ^a^
Sr (mg/kg)	12 ± 3 ^a^	20 ± 3 ^a^	15 ± 2 ^a^	7 ± 2 ^a^	6 ± 2 ^a^	8 ± 2 ^a^	11 ± 4 ^a^	10 ± 3 ^a^	13 ± 6 ^a^
Y (μg/kg)	882.84 ± 89.57 ^a^	871.83 ± 76.97 ^a^	866.40 ± 83.77 ^a^	820.49 ± 126.44 ^a^	891.47 ± 98.12 ^a^	817.53 ± 144.60 ^a^	1017.80 ± 122.24 ^a^	1020.78 ± 135.79 ^a^	1024.94 ± 145.27 ^a^
La (μg/kg)	104.81 ± 28.51 ^a^	118.79 ± 36.43 ^a^	161.04 ± 46.52 ^a^	22.65 ± 3.31 ^a^	26.91 ± 6.35 ^a^	25.70 ± 2.23 ^a^	149.27 ± 68.38 ^a^	78.72 ± 41.09 ^a^	117.96 ± 75.00 ^a^
Pr (μg/kg) *	21 ± 7 ^a^	27 ± 7 ^a^	29 ± 8 ^a^	6 ± 2 ^a^	7 ± 1 ^a^	6 ± 1 ^a^	36 ± 14 ^a^	18 ± 7 ^b^	28 ± 14 ^ab^
Nd (μg/kg) *	77.34 ± 30.11 ^a^	99.98 ± 26.08 ^a^	106.19 ± 31.11 ^a^	20.61 ± 4.17 ^a^	26.65 ± 7.40 ^a^	26.33 ± 5.63 ^a^	139.23 ± 52.80 ^a^	71.99 ± 27.79 ^b^	111.59 ± 55.50 ^ab^
Sm (μg/kg) *	16 ± 6 ^a^	20 ± 6 ^a^	21 ± 4 ^a^	5 ± 1 ^a^	7 ± 2 ^a^	7 ± 2 ^a^	30 ± 11 ^a^	16 ± 6 ^b^	24 ± 12 ^ab^
Eu (μg/kg) *	4.5 ± 0.8 ^a^	5.7 ± 2.0 ^a^	5.6 ± 0.8 ^a^	2.1 ± 0.4 ^a^	2.9 ± 0.6 ^a^	2.9 ± 1.0 ^a^	7.4 ± 2.2 ^a^	4.6 ± 1.7 ^b^	6.6 ± 2.3 ^ab^
Gd (μg/kg) *	14 ± 4 ^a^	8 ± 6 ^a^	17 ± 6 ^a^	5 ± 1 ^a^	8 ± 3 ^a^	8 ± 4 ^a^	31 ± 10 ^a^	18 ± 7 ^b^	26 ± 12 ^ab^
Tb (μg/kg) *	1252.0 ± 207.2 ^a^	864.3 ± 64.2 ^b^	1036.8 ± 192.7 ^a^	654.1 ± 115.1 ^a^	652.9 ± 127.3 ^a^	584.9 ± 145.2 ^a^	222.6 ± 66.5 ^a^	182.2 ± 100.2 ^a^	249.6 ± 196.4 ^a^
Dy (μg/kg)	12 ± 3 ^a^	14 ± 5 ^a^	14 ± 3 ^a^	4 ± 1 ^a^	6 ± 1 ^a^	7 ± 3 ^a^	24 ± 9 ^a^	15 ± 5 ^a^	21 ± 10 ^a^
Ho (μg/kg) *	2.5 ± 0.6 ^a^	3.1 ± 0.7 ^a^	3.1 ± 0.8 ^a^	0.9 ± 0.2 ^b^	1.2 ± 0.2 ^ab^	1.5 ± 0.7 ^a^	4.8 ± 1.6 ^a^	3.2 ± 1.1 ^a^	4.3 ± 1.9 ^a^
Er (μg/kg)	8 ± 2 ^a^	1 ± 4 ^a^	9 ± 3 ^a^	3 ± 1 ^a^	3 ± 1 ^a^	4 ± 2 ^a^	14 ± 5 ^a^	10 ± 3 ^a^	13 ± 5 ^a^
Tm (μg/kg)	1.0 ± 0.4 ^a^	1.7 ± 0.7 ^a^	1.3 ± 0.5 ^a^	0.3 ± 0.1 ^a^	0.4 ± 0.1 ^a^	0.5 ± 0.3 ^a^	2.2 ± 0.9 ^a^	1.4 ± 0.5 ^a^	2.0 ± 0.8 ^a^
Yb (μg/kg) *	7 ± 3 ^a^	10 ± 4 ^a^	10 ± 3 ^a^	2 ± 1 ^b^	3 ± 1 ^ab^	4 ± 2 ^a^	14 ± 4 ^a^	9 ± 3 ^a^	12 ± 5 ^ab^
Lu (μg/kg)	3.7 ± 2.7 ^a^	3.5 ± 1.1 ^a^	3.6 ± 1.7 ^a^	7.6 ± 2.2 ^a^	8.2 ± 3.4 ^a^	9.6 ± 3.1 ^a^	12.9 ± 4.8 ^a^	9.8 ± 1.7 ^a^	11.8 ± 2.6 ^a^

Data are shown as the mean ± standard deviation. ^a,b^ in the same row indicated that there are significant differences among regions at *p* < 0.05 level. * means significant difference (*p* < 0.05).

**Table 8:** Mean square of each stable isotope and element by analysis of variance.

Source of Variation	Region (R)	Age (A)	R × A	Error
DF	2	2	4	44
cc (‰)	16.162 **	0.453	0.783	0.918
δ^15^N (‰)	89.32 **	4.98	3.31	3.24
Mg (mg/kg)	940,372.990 **	214.532	16,805.581	35,097.374
K (mg/kg)	320,175.52	4,407,718.36	1,902,670.37	2,576,350.83
Ca (mg/kg)	343,041.76	441,090.52	973,706.97	223,680.31
Mn (mg/kg)	212,6241 **	45,782	38,559,311 **	88,978,814
Fe (mg/kg)	28,113 *	12,679	7124	8161
Cu (mg/kg)	12,380.3	9732.2	1244.7	5492.3
Zn (mg/kg)	77,533.4 *	48,583.2	22,130.5	21,778.1
Rb (mg/kg)	7,570,777 **	208,510	230,305	211,491
Sr (mg/kg)	231,854 **	32,516	34,624	13,801
Y (mg/kg)	178.84 **	4.56	2.25	15.11
La (mg/kg)	51.79 **	2.90	2.40	2.32
Pr (μg/kg)	2357 **	82	152	95
Nd (mg/kg)	35.45 **	1.23	2.20	1.38
Sm (μg/kg)	1466 **	56	95	61
Eu (μg/kg)	60.9 **	1.3	4.9	2.7
Gd (μg/kg)	1439 **	28	97	57
Tb (mg/kg)	2068.2 **	0.0	0.0	0.0
Dy (μg/kg)	954 **	11	48	39
Ho (μg/kg)	39.5 **	0.7	1.6	1.4
Er (μg/kg)	363 **	2	18	13
Tm (μg/kg)	9.5 **	0.0	0.5	0.4
Yb (μg/kg)	364 **	5	22	13
Lu (μg/kg)	215.4 **	3.8	5.5	11.3

* means significant effect (*p* < 0.05), ** means highly significant effect (*p* < 0.01).

**Table 9:** The external validation based on the indicator of new Pu-erh tea samples.

Region	δ^13^C	δ^15^N	Mg	Mn	Rb	La	Tb
Bangdong	−26.01	1.40	2,724,050.97	1,235,282.25	90,404.89	523.63	8.20
	−25.98	2.74	2,772,521.47	1,066,798.51	86,209.27	289.25	6.09
	−27.43	3.19	2,632,641.73	1,043,034.78	96,223.16	214.16	4.73
	−27.91	2.75	2,686,877.79	916,569.28	102,914.99	367.90	6.83
	−26.16	2.57	3,067,714.29	969,110.57	133,737.01	375.74	5.66
	−25.36	2.50	2,658,854.62	737,281.79	108,884.34	242.07	5.16
	−25.68	2.31	2,482,755.05	1,006,464.11	102,004.56	391.33	7.49
	−25.32	1.93	2,760,370.63	1,260,159.98	120,953.10	295.24	7.58
	−26.30	2.57	2,543,127.79	947,274.99	95,336.29	369.31	6.56

## Data Availability

Data is contained within the article or supplementary material.

## Supplementary Materials

The following supporting information can be downloaded at: https://www.mdpi.com/article/10.3390/foods13030473/s1, Table S1: Stable isotopes and mineral contents in Pu-erh tea from different tree ages; Table S2: Principal component analysis table of characteristic mineral elements; Table S3: Classification results of Pu-erh tea from different regions; Table S4: Stable isotope and mineral element content in Pu-erh tea samples from different regions (Tree age, variety, etc.).
