# Supplementary material for: Effects of Geographical Origin and Tree Age on the Stable Isotopes and Multi-Elements of Pu-erh Tea
Source: Foods. 2024 Feb 2;13(3):473. doi: 10.3390/foods13030473 (PMC10855849; doi:10.3390/foods13030473)
Supplement: Supplementary file 1 [file foods-13-00473-s001.zip › foods-2800945-supplementary.pdf]

Table S1. Quality control for determining the elements of Pu-erh tea

Element	Isotope	LOD (μg/kg)	LOQ (μg/kg)	Certified value (μg/g)	Exptl value (μg/g)	Recovery (%)
Mg	24	1.485	4.454	1800.000±100.000	2154.253±175.984	120±8
K	39	1.168	3.505	14500.000±500.000	19069.778±230.292	132±1
Ca	43	0.000	0.000	4700.000±200.000	4772.470±93.162	102±2
Mn	55	0.077	0.230	1250.000±40.000	1324.841±26.427	105±2
Fe	56	0.048	0.143	149.000±7.000	140.731±6.046	94±4
Cu	63	0.013	0.039	8.300±0.500	7.422±0.066	89±1
Zn	66	0.443	1.328	27.000±3.000	26.686±0.129	99±0
Rb	85	0.041	0.124	45.900±3.300	54.023±1.027	117±2
Sr	88	0.079	0.237	13.700±0.700	15.947±0.050	116±0
Y	89	0.000	0.000	2.000±0.200	2.178±0.024	109±1
La	139	0.000	0.000	1.010±0.080	1.100±0.008	109±1
Pr	141	0.000	0.000	0.160±0.000	0.193±0.005	121±2
Nd	146	0.000	0.000	0.660±0.020	0.705±0.008	107±1
Sm	147	0.000	0.000	0.120±0.020	0.135±0.003	112±2
Eu	153	0.000	0.000	0.027±0.000	0.026±0.001	97±4
Gd	157	0.000	0.000	0.140±0.020	0.140±0.003	100±2
Tb	159	0.001	0.004	0.021±0.002	0.022±0.000	102±1
Dy	163	0.000	0.000	0.130±0.020	0.135±0.003	104±2
Ho	165	0.000	0.000	0.028±0.004	0.029±0.001	105±2
Er	166	0.000	0.000	0.080±0.010	0.091±0.001	114±1
Tm	169	0.000	0.000	0.014±0.002	0.015±0.001	109±4
Yb	172	0.000	0.000	0.100±0.020	0.101±0.002	101±2
Lu	175	0.000	0.000	0.016±0.003	0.017±0.001	106±3

Table S2. Principal component analysis table of characteristic mineral elements

Element	Component			
	1	2	3	4
$\delta^{13}C$ (‰)	0.722	0.087	0.032	0.449
$\delta^{15}N$ (‰)	0.446	0.641	0.104	0.072
Mg (mg/kg)	-0.557	-0.177	0.629	-0.275
Fe (mg/kg)	0.530	-0.699	0.090	0.192
Cu (mg/kg)	0.103	0.456	0.387	0.535
Rb (μg/kg)	-0.381	-0.119	0.283	0.353
Sr (μg/kg)	0.562	0.511	-0.275	0.205
Y (μg/kg)	0.267	0.565	-0.642	-0.080
La (μg/kg)	0.679	-0.348	-0.009	0.328
Pr (μg/kg)	0.855	0.264	0.285	-0.107
Nd (μg/kg)	0.925	0.193	0.195	-0.156
Sm (μg/kg)	0.946	0.150	0.174	-0.145
Eu (μg/kg)	0.966	0.058	0.157	-0.128
Gd (μg/kg)	0.953	0.049	-0.011	-0.143
Tb (μg/kg)	0.973	-0.076	0.117	-0.098
Dy (μg/kg)	-0.442	0.659	0.388	-0.121
Ho (μg/kg)	0.975	-0.133	0.051	-0.069
Er (μg/kg)	0.978	-0.078	-0.001	-0.031
Tm (μg/kg)	0.979	-0.048	-0.011	-0.032
Yb (μg/kg)	0.959	-0.061	-0.016	-0.017
Lu (μg/kg)	0.970	-0.031	-0.015	-0.025
Variance contribution rate (%)	57.758	13.427	6.756	4.595
Cumulative variance contribution rate (%)	57.758	71.185	77.941	82.536

Table S3. Classification results of Pu-erh tea from different regions

		Geographical Origin	Prediction group Member Information			Total
			Jinggu	Bangdong	Ning'er	
Original	Counts	Jinggu	24	0	0	24
		Bangdong	0	14	0	14
		Ning'er	0	0	15	15
	%	Jinggu	100.0	0	0	100.0
		Bangdong	0	100.0	0	100.0
		Ning'er	0	0	100.0	100.0
Cross-validation[b]	Counts	Jinggu	24	0	0	24
		Bangdong	0	14	0	14
		Ning'er	0	0	15	15
	%	Jinggu	100.0	0	0	100.0
		Bangdong	0	100.0	0	100.0
		Ning'er	0	0	100.0	100.0

Note: 100.0% of the initial grouping cases have been correctly classified

Table S4. Stable isotope and mineral element content in Pu-erh tea samples from different regions (Tree age, variety, etc).

Region	Bangdong													
Tree Age (year)	20~100	20~100	20~100	20~100	20~100	20~100	>200	20~100	100~200	>200	100~200	>200	20~100	100~200
Sample	1	2	3	4	5	6	7	8	9	10	11	12	13	14
δ13C(‰)	-24.85	-25.21	-25.82	-24.82	-23.37	-25.24	-24.11	-24.32	-25.72	-25.6	-25.56	-26.29	-26.29	-25.09
δ15N(‰)	-0.08	1.61	1.68	-0.05	1.48	0.43	4.12	7.37	10.59	4.71	4.52	7.27	7.8	5.34
Mg (mg/kg)	1933.790926	1377.636056	2071.673797	1938.261961	1744.924296	1884.516916	1648.625157	1751.593855	1905.679904	1940.803608	1751.277029	1673.637547	1705.79323	1538.246473
K (mg/kg)	22793.06339	18507.46679	19596.39126	19277.19449	18623.49796	22234.75158	17985.19298	18871.40501	18796.22461	20042.90078	18476.96275	19364.51893	18057.2528	22022.56182
Ca (mg/kg)	4511.097518	5630.1373	4262.72897	3926.62344	5134.004873	4622.713157	3782.16298	4030.618735	4414.50676	3820.892963	2660.417534	4767.659043	4472.090853	2676.118999
Mn (mg/kg)	283641.7987	551823.9362	512484.2375	186330.3624	329678.8264	220928.2388	349203.0366	471454.6334	482459.92	421775.2481	202096.0353	559101.2056	199855.2371	486306.5685
Fe (mg/kg)	114.879033	118.0653807	121.3967054	131.1000437	91.68076095	103.6500869	83.12540677	699.248704	71.77873574	136.9254637	256.793849	79.58556762	72.99306196	85.80994289
Cu (mg/kg)	17811.9336	12782.76031	9889.450887	12352.38765	11920.66496	12419.33251	13457.58146	10386.64432	11873.4961	12304.50672	17915.84612	11712.21499	9390.739661	13605.17784
Zn (mg/kg)	41236.28268	30088.06348	37300.23226	36520.31499	41021.57334	37367.45279	36706.20436	31840.6528	45441.19661	30558.10163	35645.78846	30016.1894	28687.77158	23551.7036
Rb (mg/kg)	73453.2925	119775.5295	65516.72676	64525.44136	49166.32472	79403.9343	102842.0035	90720.33778	86231.87592	73908.76049	60524.06914	69415.10308	71519.80266	38633.48908
Sr (mg/kg)	12466.23064	15666.11939	8066.538584	12426.94641	14282.60947	15258.32205	16480.18956	7171.625313	21217.74645	14977.86779	16091.20331	13054.52566	7980.98688	22528.93108
Y (μg/kg)	790.7103045	768.3087831	985.1652807	923.9669574	924.4012306	944.1050379	963.0877543	952.8166865	791.3616135	820.3676579	879.4053186	815.7454474	773.2493043	944.7324536
La (μg/kg)	121.7436228	81.50891618	88.32477682	87.83589142	73.1151545	106.4572196	212.7466445	119.1518836	152.1577339	122.5932065	79.92979538	147.7864862	160.3725838	124.2717853

Region	Ning'er														
Tree Age (year)	20~100	100~200	>200	20~100	100~200	>200	20~100	100~200	>200	20~100	100~200	>200	20~100	100~200	>200
Sample	1	2	3	4	5	6	7	8	9	10	11	12	13	14	15
δ13C(‰)	-26.58	-26.12	-27.65	-27.32	-26.78	-27.61	-26.88	-25.42	-27.24	-26.74	-26.59	-26.6	-27.31	-26.04	-26.66
δ15N(‰)	-0.41	-0.98	-0.3	-0.97	0.22	0.05	0.29	0.74	-0.42	-0.77	1.32	0.55	-0.8	-0.23	0.03
Mg (mg/kg)	1799.317459	2179.865754	1909.465717	2306.994678	1947.715651	2183.755723	2049.617737	2243.362011	2088.633184	1881.329531	2042.062667	2059.961904	2075.448347	2320.508779	2070.762072
K (mg/kg)	19566.40932	19317.75606	18625.18009	20007.24846	19287.03775	19956.85442	18570.4647	22666.75127	19300.82689	19343.97412	20277.90861	19860.0377	20713.39263	19592.06153	18432.83323
Ca (mg/kg)	3397.332503	4657.043448	4522.530085	4513.140319	4207.709288	4276.620396	3878.083548	5045.286083	3268.714862	4021.618473	4204.102977	4118.724258	3621.238373	4548.442581	4479.423473
Mn (mg/kg)	1028199.488	748242.3729	737063.3463	473657.1821	929744.9283	925822.3969	649337.946	735126.2762	1040784.13	439122.5443	935602.5007	490299.3526	683153.72	1150510.484	892997.1785
Fe (mg/kg)	54.76978753	64.48249005	67.58598541	60.57852712	57.37559594	61.11166363	59.37096216	65.07268587	66.74212265	144.877625	66.73457118	71.77591899	60.84201889	77.24277158	71.67667417
Cu (mg/kg)	16599.211	16358.32364	14164.88919	15675.04969	14490.88489	13390.38401	14886.65761	14997.84547	14108.831	16618.38842	15908.33549	15879.37273	14189.49768	16209.73743	14415.6685
Zn (mg/kg)	42874.35743	40512.7417	28131.67931	33504.28636	40598.43554	29636.13613	36366.2398	44074.08394	35341.45372	37869.89295	43970.23882	38184.1682	34964.75517	39400.44851	33924.76711
Rb (mg/kg)	34939.41396	34858.68502	35785.55482	22108.46738	24151.57986	33791.78567	24376.23789	22557.09234	28831.69291	23270.33383	16656.56098	24682.04419	23192.72457	36046.74555	35078.35066
Sr (mg/kg)	4244.469267	8810.721135	9914.751313	8158.646069	4517.877657	7793.226808	5892.213808	5516.081093	3601.555935	7837.115283	4740.076233	7886.257299	6463.457467	4083.534318	8284.707474
Y (μg/kg)	783.7410668	764.9035889	809.0320745	680.0069754	847.5554893	731.0525882	999.8664282	1019.390233	889.5090619	746.3103407	872.1962263	1016.962708	892.509186	953.3121733	641.1026121
La (μg/kg)	19.86673833	29.93636542	28.51975229	26.43462893	25.26410574	24.59463407	19.14552927	22.46342709	27.55859271	22.09794713	20.53373434	23.2263009	25.68281318	36.34053946	24.59095972

Region	Jinggu																							
Tree Age (year)	>200	100~200	20~100	20~100	>200	100~200	20~100	20~100	>200	20~100	20~100	100~200	>200	20~100	100~200	20~100	>200	100~200	20~100	20~100	>200	100~200	20~100	20~100
Sample	1	2	3	4	5	6	7	8	9	10	11	12	13	14	15	16	17	18	19	20	21	22	23	24
δ13C(‰)	-24.65	-24.49	-25.75	-23.88	-24.65	-24.74	-24.12	-24.24	-23.51	-23.41	-24	-22.72	-24.38	-25.92	-25.8	-26.41	-26.69	-26.73	-24.08	-25.44	-26.34	-26.05	-23.26	-24.44
δ15N(‰)	5.28	2	3.02	2.28	1.07	0.36	0.13	1.24	4.44	2.69	4.5	2.26	2.26	2.28	2.55	2.27	2.28	2.79	4.84	1.88	1.66	2.5	1.46	1.39
Mg (mg/kg)	1750.769847	1269.082584	1848.340342	1903.918368	1532.53473	1805.762086	1542.554236	1764.407457	1804.25836	1447.937657	1507.336881	1667.789697	1521.337976	1497.684826	1786.124113	1538.130784	1610.82487	1436.064114	2030.344352	1653.000868	1516.986759	1294.686235	1300.956097	1414.449369
K (mg/kg)	18433.51369	18714.74214	20397.65196	23036.4435	19274.01907	18995.26866	21881.24109	18919.59613	18723.51002	18995.15222	18309.82585	19785.97115	18025.63376	24248.52605	20462.70618	21506.83336	22335.41458	19587.98994	22301.55117	19278.71315	23042.82992	18910.87843	20792.36457	22891.82474
Ca (mg/kg)	4050.902728	4009.90788	3878.70764	3730.730642	4098.255406	3206.018238	3418.067114	4349.361489	4862.076088	4188.606727	3426.355723	3977.408109	4199.157464	4193.559622	3181.476693	4375.406563	3697.476222	3070.604152	4633.798378	3205.98888	3978.623745	3708.499639	3693.272091	3619.026411
Mn (mg/kg)	1305565.043	1437580.22	1412908.147	1463254.715	1170486.194	1190763.466	747865.9858	1968000.465	1855367.316	1449264.931	1472836.164	1475533.512	650993.4174	1025273.368	724887.4379	1072103.08	823274.3057	880987.2198	1226960.379	743199.6518	750980.9598	1068635.351	864087.139	1037679.85
Fe (mg/kg)	85.11186528	71.19908873	115.6590796	136.4414517	87.77467664	72.46835639	83.27340953	95.9591997	101.7003047	108.8277611	76.70443647	90.20514703	62.37330721	64.56524914	67.94142487	63.89547943	62.73682672	85.83582919	119.226692	67.78154924	77.33946003	68.59858253	80.6682689	68.91649485
Cu (mg/kg)	14119.20879	21269.15602	15845.25395	14527.15489	97918.33943	13032.05752	10237.70252	14824.85049	16364.37639	16207.82319	14129.58098	12399.52	12595.85536	12610.98531	13583.75101	11803.68573	12923.80838	14293.41889	15614.15731	12565.72718	12858.41565	16273.23871	17155.53873	18087.99696
Zn (mg/kg)	29144.36002	35112.5211	43644.39983	32054.70515	30618.8108	31315.31302	30303.56475	34586.89197	33890.36286	38992.51995	31835.12336	33108.11517	39947.58564	30656.12842	36023.82834	29923.36349	26812.78136	38320.15088	28357.65305	27707.9257	30762.88217	32876.9669	33963.59573	29410.35973
Rb (mg/kg)	34442.77675	54287.91117	60888.8201	68876.97456	37602.89694	42311.15364	38487.9516	81138.69583	58619.65699	55252.65671	56857.42249	69558.18032	65376.71875	92183.94725	67612.87965	96997.53912	80027.57092	86025.74119	50893.14032	63956.68334	85042.80614	73068.66883	66367.6331	73612.15124
Sr (mg/kg)	6514.184216	13612.66398	6616.129098	7926.351394	9586.316163	8115.216106	10078.50991	9655.880469	8841.528295	8033.29701	5851.589272	6798.80226	24002.0496	16933.79994	7976.610263	12270.57743	14051.74459	11769.80151	7321.80847	12898.07421	16515.85673	14257.60787	15048.40515	15063.84138
Y (μg/kg)	976.6633185	1239.393918	994.4032164	892.2201439	965.3280516	1103.538364	993.5208899	1268.50351	1307.636083	942.8419303	1055.654705	1036.196534	919.5721514	851.5548184	965.0447317	1000.85165	934.5213101	898.5464309	1164.452828	993.936398	1045.895181	881.9717765	910.8411144	1144.78268
La (μg/kg)	203.8673585	100.3926271	209.2155228	204.0288712	137.3962615	113.4066577	105.9701007	182.9986855	204.880775	134.0797982	93.20442103	131.4533332	46.63354483	112.3121415	46.03785409	63.88578079	43.20634757	39.86392087	290.7382269	81.01847763	71.77857956	41.15678294	206.9004718	106.8387244

Pr	20.7 17.1 18.7 17.0 12.2 16.5 38.5 28.3 30.0 23.2 19.0 25.6 34.5 31.1 4.64 7.58 6.14 9.84 6.46 5.32 4.39 6.09 8.15 4.24 5.44 4.25 6.31 8.77 5.82 43.7 26.5 50.1 43.0 28.9 23.2 25.3 40.8 40.8 26.8 19.1 24.6 9.94 38.0 13.5 15.3 13.3 10.8 65.5 26.4 29.6 10.3 44.7 34.1
(μg/k	09517230248352227353056881661689357767232465063205007748760719005383647345402994896122451093833612193024645310515942644548441352056678244081960829350975315168123169061602931901460833604900738994880535010355936376
g)	199 522 521 493 379 477 934 212 108 992 211 667 424 18 292 084 125 65 309 596 674 038 075 777 863 66 428 721 801 569 808 64 25 423 032 742 682 106 408 101 12 564 121 66 488 827 742 05 295 939 251 197 531
Nd	75.6 62.3 70.2 62.8 44.8 57.7 141. 109. 115. 83.4 69.8 93.4 135. 114. 18.1 27.3 27.4 22.4 20.7 24.7 16.1 24.2 35.2 19.4 21.7 20.0 26.8 39.0 24.1 171. 107. 193. 164. 111. 89.6 102. 167. 163. 108. 69.6 92.4 38.6 146. 56.7 61.1 53.5 43.7 244. 103. 130. 42.4 163. 145.
(μg/k	94499448357352426952240064503168451659446715607565226295291263165028574890057751904015871871229996356922644289833647712304072698965004630065390882287047278907210507123511585882363548890821544883579241202707207539
g)	776 551 099 17 273 939 234 353 53 973 639 24 98 281 843 368 342 758 006 376 209 456 393 012 408 435 601 943 304 907 904 294 665 585 502 583 171 191 556 704 042 019 364 828 368 348 873 552 431 897 443 474 254
Sm	15.0 12.8 15.9 11.9 10.9 11.1 25.5 24.6 22.1 17.9 12.7 18.7 25.5 24.6 5.12 6.06 7.78 4.52 6.36 7.56 3.59 5.09 10.5 5.07 5.05 5.30 7.54 10.4 5.12 32.9 23.4 42.2 33.5 26.6 21.5 24.8 41.3 35.2 22.5 16.6 19.7 7.74 31.4 13.7 12.5 11.8 9.13 49.6 21.1 31.6 8.71 33.7 34.3
(μg/k	19476691419563212648645936517345317739621801661111154392192917259758786112653814093538741531315476888301625356463186411529177202361722241016929489642071622503633924784838907506345361019384035340326781838317636123
g)	292 009 617 759 757 134 077 234 959 963 101 631 048 415 024 49 549 724 887 274 69 085 142 661 172 69 882 771 215 996 378 171 04 896 634 019 49 545 971 005 298 485 757 447 393 893 612 53 172 644 337 053 364
Eu	5.16 4.85 3.83 3.94 3.44 3.98 6.54 4.81 6.11 5.32 3.59 4.92 5.88 7.46 1.96 3.35 3.55 1.95 2.39 2.03 2.03 2.31 4.26 2.91 2.85 2.06 1.80 3.67 2.58 7.54 7.83 8.81 7.14 5.70 5.02 6.40 10.8 9.51 5.03 4.24 4.58 3.69 8.45 3.88 4.25 4.84 3.12 9.18 6.03 8.47 3.30 9.31 8.51
(μg/k	34977699424630955787891915053308483389851101821941378053453596631858306884696042564614451360459285482638070055247839288258771714725135457543727036517679485084773313277632338509526992950468841893373222255313219369
g)	01 93 883 527 393 665 818 33 071 151 898 419 288 074 657 239 925 293 759 967 484 368 109 581 003 384 798 558 914 509 718 73 129 523 357 912 468 12 654 974 153 478 721 399 788 626 747 792 829 842 679 695 897
Gd	14.9 12.8 14.9 11.7 9.24 10.0 23.9 19.1 22.3 12.7 11.0 13.0 20.2 20.8 5.92 7.46 7.62 5.17 6.91 7.15 5.18 8.12 15.0 4.65 4.65 5.86 6.47 13.2 5.43 32.5 25.6 36.8 36.1 26.0 23.8 25.3 45.1 37.6 23.2 17.4 19.1 9.59 29.9 15.9 16.1 14.6 11.4 48.0 21.2 35.2 9.35 34.7 32.2
(μg/k	05076850464294593012853289672236611196243143210751294207648308291100037832578624983146821252831426692679289316016750835198912631086272037381593302272083343208047829029455542969291869052837161082145674883998469650
g)	163 48 054 899 504 383 242 771 341 708 974 943 14 348 529 861 792 501 952 267 112 496 263 252 206 505 172 058 615 901 129 702 508 526 416 063 121 629 292 081 554 424 967 878 771 175 441 075 21 176 723 324 953
Tb	1144142313901366143113551256999. 887. 895. 914. 958. 904. 791. 809. 706. 497. 630. 793. 817. 723. 726. 626. 514. 539. 534. 592. 498. 448. 539. 313. 292. 279. 358. 239. 319. 178. 132. 272. 227. 185. 58.7 213. 112. 111. <0.0 <0.0 124. 215. 159. 60.6 264. 171.
(μg/k	.848 .419 .561 .523 .229 .823 .283 24351768420901086085628584259523884895614370543424493929227917655038571118273673415481236057765881498859177713069596457026502299230703522461399062175589 00 00 169371621836599627531363
g)	089 167 412 819 001 97 554 55 874 055 895 034 746 581 042 145 852 868 947 69 586 428 84 182 778 871 456 398 436 158 366 112 065 992 871 018 966 894 342 3 763 347 85 411 178 66 963 492 419 96 465
Dy	12.0 13.5 15.5 11.7 8.11 6.61 17.4 13.9 16.1 12.3 8.44 11.2 14.2 18.6 6.33 5.34 4.85 4.24 5.92 6.44 3.13 5.92 12.6 3.77 4.60 5.97 4.49 8.20 5.09 22.2 22.3 27.1 24.7 20.4 18.2 21.1 43.0 33.0 17.8 13.9 16.3 5.60 22.9 16.0 15.0 15.3 9.85 35.8 17.2 27.5 9.99 25.7 27.6
(μg/k	47789154062709428781041285753056186824989864467070434532850996248589365209446354528898798633191291372691013706153444981498917457899692585170174106958305209119424903322381393902841526747291940882654940516158589031
g)	689 796 033 375 823 085 85 495 211 555 277 576 311 587 126 199 185 383 732 952 714 893 166 972 742 686 419 295 529 089 599 164 468 691 178 611 584 544 579 444 383 192 436 458 295 461 318 172 753 34 112 12 187
Ho	2.27 2.71 3.01 2.12 1.78 1.61 3.92 3.18 3.25 2.88 2.30 2.34 3.28 3.69 1.14 1.06 1.12 0.73 1.35 1.39 0.88 1.03 2.67 0.57 0.90 1.26 0.90 1.47 0.97 4.37 4.70 5.33 4.17 4.23 3.68 4.21 8.07 6.64 3.88 2.94 3.70 1.61 4.75 3.04 2.93 2.90 2.19 6.55 3.35 5.86 1.83 4.91 6.67
(μg/k	33434600734268323810500488779163225111734209558954575127672574419526191454093085903295846759566051152512591558997145312548768430554187685170705606436946921343537680908178717499179430254142148953903391149382781976
g)	831 408 979 68 192 33 058 707 079 03 108 895 123 546 765 157 374 882 183 308 055 877 577 33 545 905 461 809 999 884 667 739 702 442 255 332 173 948 96 482 404 938 586 332 002 768 428 492 262 861 567 936 167
Er	7.54 9.68 9.95 5.93 5.95 5.01 12.6 9.35 11.6 7.84 6.03 7.58 9.55 13.9 3.42 2.88 3.42 2.03 3.44 3.75 2.42 3.59 7.79 1.88 3.09 2.50 2.81 4.33 2.97 12.8 13.1 13.6 14.8 13.8 11.0 12.6 25.6 19.5 12.1 8.87 11.0 4.59 12.3 9.44 9.74 10.0 6.33 19.3 11.3 15.8 6.55 13.8 18.9
(μg/k	11144694196329362327567249625132779042336057064553504338616643668123311131958588911568613681414751464571214335888634060995581617574850360101113127407678524702855085564741593138511999001282874264823410791784371449
g)	477 389 436 275 186 966 959 703 906 832 708 403 974 724 722 543 462 921 084 857 335 033 879 175 533 185 046 074 203 784 5 194 062 171 351 594 4 232 222 87 918 377 027 266 957 109 45 965 277 07 394 512 931
Tm	0.88 1.37 1.34 0.77 0.53 0.39 1.87 1.45 1.92 0.84 0.90 1.16 1.52 2.19 0.46 0.26 0.27 0.25 0.49 0.60 0.26 0.46 1.04 0.28 0.21 0.32 0.29 0.44 0.38 1.91 2.14 1.78 2.13 2.04 1.72 2.04 4.31 3.14 1.92 1.31 1.63 0.60 1.78 1.34 1.25 1.74 0.89 2.91 1.63 2.44 0.87 1.77 3.06
(μg/k	151043082569 6844 3827935751374233871254619343476557526548605068128342155780109456924004955687746415889201163502545584940621384208509592456536445237365477154308173307523869820898055080378799730525485756621341340741
g)	569 038 592 046 254 797 317 79 632 421 183 756 994 411 599 467 617 853 399 745 122 004 322 928 466 076 657 124 438 426 343 657 428 376 22 442 807 972 247 355 616 795 407 965 254 996 115 645 662 156 589 5
Yb	5.27 8.91 7.99 5.29 4.61 3.16 13.3 9.30 10.3 9.79 5.62 6.98 9.82 13.7 2.70 2.77 2.73 2.08 3.30 4.06 1.41 3.80 6.27 1.60 2.14 2.59 1.74 3.81 2.28 12.1 13.5 12.7 16.6 12.8 11.4 14.3 23.8 19.7 12.0 8.89 10.1 4.02 12.0 9.12 7.81 10.3 6.28 16.4 12.2 13.6 5.02 14.8 19.3
(μg/k	69460124107078181643251287655642322100495094702112685709355823617145705321679824018894597488781660359078897658018666549383219969721497582551075799991259210325538534841759946384035263858937884635563493886299776996
g)	393 463 026 283 461 257 815 324 474 712 078 514 447 712 07 132 301 577 848 015 39 834 83 941 977 026 923 022 357 928 102 825 966 989 235 04 909 36 87 067 995 912 488 189 652 482 588 478 078 718 718 553 764
Lu	6.20 <0.0 <0.0 <0.0 <0.0 0.24 4.67 2.88 4.43 1.59 3.68 4.53 5.32 2.31 5.09 6.56 11.2 6.17 3.77 11.7 10.0 8.53 6.11 9.80 13.0 12.4 7.02 9.21 6.19 10.9 8.39 16.9 12.3 10.7 10.5 13.3 21.1 16.9 11.9 5.54 9.79 11.6 17.5 12.1 15.0 10.3 7.18 13.0 6.20 9.97 10.4 6.78 15.2
(μg/k	9530 00 00 00 00 149662831171287711065287435929124343009426642588825306614063646749272939826154355835986436731982487222293829943938264201846671741810131834573711200555312392505314248952623711370560803994811029
g)	462 267 377 428 068 529 369 815 918 981 635 885 642 707 623 764 906 386 577 247 899 504 746 86 026 622 79 864 234 147 353 144 694 5 314 314 395 49 873 979 261 229 029 912 785 738 756 287 702
